# Landscape and Regional Environmental Analysis of the Spatial Distribution of Hantavirus Human Cases in Europe

**DOI:** 10.3389/fpubh.2015.00054

**Published:** 2015-03-31

**Authors:** Caroline Brigitte Zeimes, Sophie Quoilin, Heikki Henttonen, Outi Lyytikäinen, Olli Vapalahti, Jean-Marc Reynes, Chantal Reusken, Arno N. Swart, Kirsti Vainio, Marika Hjertqvist, Sophie O. Vanwambeke

**Affiliations:** ^1^Georges Lemaître Centre for Earth and Climate Research, Earth and Life Institute, Université Catholique de Louvain (UCL), Louvain-la-Neuve, Belgium; ^2^Epidemiology Unit of Infectious Diseases, Scientific Institute of Public Health, Brussels, Belgium; ^3^Natural Resources Institute Finland, Vantaa, Finland; ^4^Infectious Disease Control Unit, Department of Infectious Diseases, National Institute for Health and Welfare, Helsinki, Finland; ^5^Infectious Disease Control Unit, Department of Virology, University of Helsinki, Helsinki, Finland; ^6^Unité de Biologie des Infections Virales Emergentes, Centre National de Référence des Hantavirus, Institut Pasteur, Lyon, France; ^7^Department of Viroscience, ErasmusMC, Rotterdam, Netherlands; ^8^Centre for Infectious Disease Control, Rijksinstituut voor Volksgezondheid en Milieu (RIVM), Bilthoven, Netherlands; ^9^Department of Virology, Norwegian Institute of Public Health, Oslo, Norway; ^10^The Swedish Institute for Infectious Disease Control, Stockholm, Sweden

**Keywords:** hantavirus, environmental modeling, spatial model, multilevel logistic regression, large scale model

## Abstract

**Background:** In Europe, the most prevalent hantavirus, Puumala virus, is transmitted by bank voles and causes nephropathia epidemica in human. The European spatial distribution of nephropathia epidemica is investigated here for the first time with a rich set of environmental variables.

**Methods:** The influence of variables at the landscape and regional level is studied through multilevel logistic regression, and further information on their effects across the different European ecoregions is obtained by comparing an overall niche model (boosted regression trees) with regressions by ecoregion.

**Results:** The presence of nephropathia epidemica is likely in populated regions with well-connected forests, more intense vegetation activity, low soil water content, mild summers, and cold winters. In these regions, landscapes with a higher proportion of built-up areas in forest ecotones and lower minimum temperature in winter are expected to be more at risk. Climate and forest connectivity have a stronger effect at the regional level. If variables are staying at their current values, the models predict that nephropathia epidemica may know intensification but should not spread (although southern Sweden, the Norwegian coast, and the Netherlands should be kept under watch).

**Conclusion:** Models indicate that large-scale modeling can lead to a very high predictive power. At large scale, the effect of one variable on disease may follow three response scenarios: the effect may be the same across the entire study area, the effect can change according to the variable value, and the effect can change depending on local specificities. Each of these scenarios impacts large-scale modeling differently.

## Introduction

In Europe, Puumala virus (PUUV), the most prevalent hantavirus in Western and Northern Europe (1), is responsible for hemorrhagic fever with renal syndrome, often called nephropathia epidemica (NE) in humans (2, 3). The reservoir is the bank vole (*Myodes glareolus*) (4). Transmission to humans may be direct, by biting, but is mainly indirect, by inhalation of dust of urine and feces of infected bank voles. For example, while cleaning closed and un-aired buildings or handling firewood (5). Transmission between bank voles is also both direct and mainly indirect (6–8).

Nephropathia epidemica cases have been recorded in many European countries. A number of studies have investigated the environmental factors influencing PUUV distribution, but never more broadly than at the national level [e.g., Ref. (9, 10)]. While the main environmental factors are fairly well known, and hypotheses explaining the broad distribution of PUUV exist (2, 11, 12), no study so far has investigated the distribution of NE cases in humans or in bank voles at the continental scale. This is attempted here for Belgium, Finland, France, the Netherlands, Norway, and Sweden with a rich set of environmental variables related to climate, land use, vegetation, soil, and human distribution. A broad range of conditions and diverse environments are encountered across Europe and various factors identified from the literature may be more relevant for some or other areas. Among climatic factors, higher humidity and cold temperatures favor *ex vivo* survival (8, 9). However, higher winter temperature may relate to more human outdoors activities and a higher exposure to bank vole excreta (13). In Fennoscandia, a thick snow cover shelters bank voles against predators and, in the absence of an ice sheet, preserves food for the bank voles (14–18). Snow also protects the virus from UV light and maintains the preferred stable cold temperature and high humidity for the *ex vivo* virus survival (8). In areas where snow cover is often abundant, higher winter temperatures may also lead to more contacts between human and bank voles migrating near dwellings because of the lack of protective snow cover (19, 20). In areas where the snow cover is generally low, extreme cold winter condition may also force bank voles to migrate near human dwellings (21). In areas where the snow cover is too abundant (more than 120 days/year), there is a lower prey diversity and more pressure on bank voles (14, 16, 17, 22). Land use is also important. Wet environments are preferred over drier environments (23). Forest is the main habitat of the bank vole and broadleaved forests are assumed to be the most preferred type (24). Coniferous mature and moist forests with well-developed undergrowth can also be favorable (25). Contiguous, well-connected forests may favor virus circulation in rodents (26). Buildings within 50 m of forests may lead to an increased human exposure (27). Concerning the vegetation, extended vegetation growing period increases the number of breeding opportunities for bank voles by enhancing the carrying capacity (28). Finally, soil moisture (related among other elements to soil texture) may imply a better *ex vivo* survival (8), and more humans mean higher probability of human infections.

Ecological processes take place at diverse scales, ranging from the planet, continental, regional, landscape, and local ecosystem level (29). Transitions between scales may not follow a gradual change and, in this nested structure, processes encountered at various scales differ. Looking at a broad study area means that environmental factors operating at various scales need to be considered. Our first objective is to understand the level at which variables operate and how they influence PUUV distribution. The effect of the environmental factors is tested jointly at the landscape and regional scale, using multilevel models.

Provided a regional structure is identified, investigating in further detail the regional and local effects of environmental factors are valuable in understanding the spatial distribution of NE cases across Europe. Our second objective is to understand what the effects of environmental variables are across diverse European ecoregions. This is done by using both logistic regressions on ecoregions and overall niche modeling.

## Materials and Methods

### Data

#### Disease record collections

Data are collected for Belgium, Finland, France, the Netherlands, Norway, and Sweden. As recording protocols and time span of data availability vary across countries, the study focuses on presence or absence locations. At least 5 years of records are available for the countries included. Presence record is included in the database when spatial information is provided. In France, NE cases from 2003 to 2012 are reported by municipality of exposure (473 records) or by municipality of residence (289 records), and 192 records had no spatial information (Centre National de Référence des Hantavirus, Institut Pasteur). In Belgium, NE cases from 2000 to 2010 are reported by municipality of residence (Institut scientifique de Santé publique-Wetenschappelijk Instituut Volksgezondheid). In the Netherlands, official notifications for 2008–2012 are provided by municipality of residence [Rijksinstituut voor Volksgezondheid en Milieu (RIVM)]. This dataset is completed with data from a cross-sectional population serological survey of 2929 persons sampled in 2006 (RIVM) and with the RIVM diagnostic database (RIVM is one of three diagnostic centers) for 2007–2011. In Norway, cases reported from 1989 to 2012 are provided by municipality of residence (Folkehelseinstituttet). In southern Sweden, human infections reported for 1997–2012 are collected by municipality of infection (Smittskyddsinstitutet). In northern Sweden, 212 human infections by coordinates of infection are recorded for 1991–1998 (Smittskyddsinstitutet). In Finland, the National Institute of health and Welfare collects the data that are generally available at hospital district level (province level). The coordinates of the administrative unit center were recorded for 2004–2009 (Metsäntutkimuslaitos). For this research, some high-incidence and low-incidence regions are selected.

**Figure 1:**
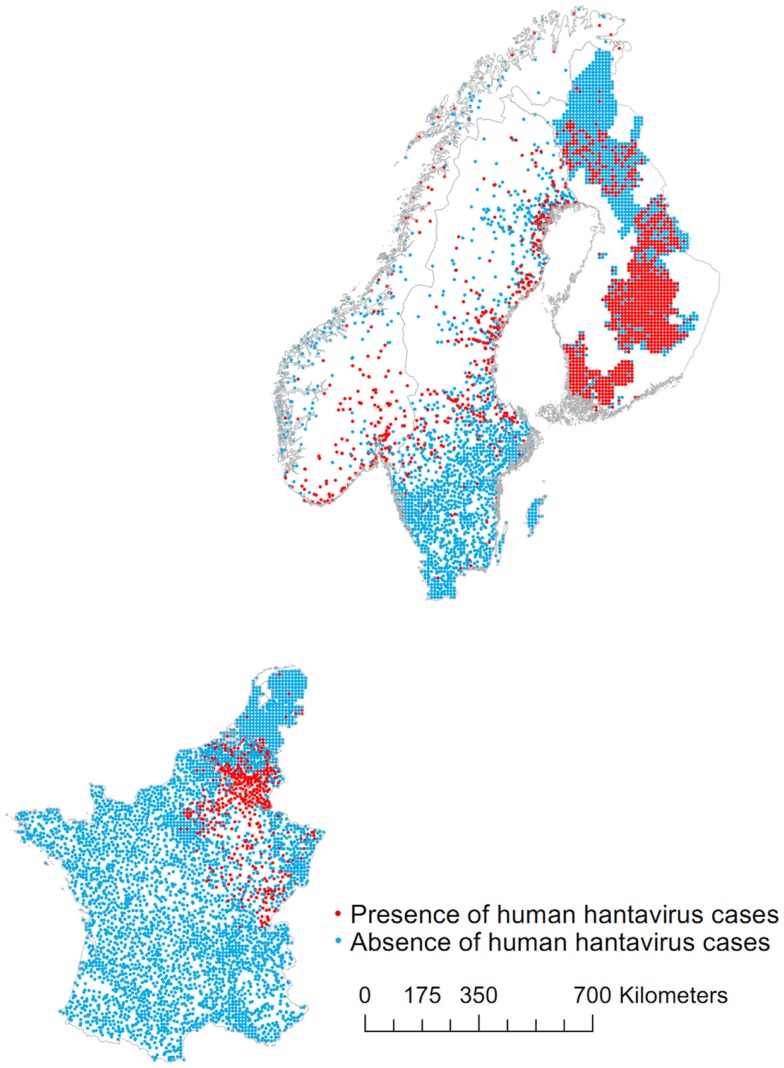
**Map of presences and absences of human hantavirus cases in Belgium, Finland, France, the Netherlands, Norway, and Sweden**.

The map of presences and absences is presented in Figure 1. While the resolution is homogeneous across the data (municipality for most countries), the average size of this unit varies greatly. In order to accommodate this, the data are converted into points locations in the following way. The centroid of municipalities between 80 and 150 km^2^ is used as the absence/presence point. Small municipalities (area ≤80 km^2^) are aggregated in a 10 km resolution grid of points. Larger municipalities (area >150 km^2^) are represented by points located where the population density is highest (2.5 arc-min resolution, Gridded Population of the World from Center for International Earth Science Information Network). Geographical coordinates are used when available. In Finland, the presence and absence data are aggregated on a 10 km grid of points. In northern Sweden, 300 absences are randomly selected among other dwellings [see Ref. (10)]. There are a total of 1933 presences and 6425 absences.

#### Environmental variables

Relevant environmental variables are identified based on a literature review of PUUV ecology in Europe, as summarized in Table 1. As this study focuses on the spatial distribution, seasonal or yearly temporal fluctuations are ignored and values are averaged over time. Two ecologically relevant levels are identified for the multilevel analyses: the landscape (individual) and the region (group). For operational purposes, the landscape level is defined as the area covered by a circular radius of 5 km around a presence/absence record. A sample size of at least 50 groups is recommended for a good estimation of regression coefficients in multilevel regressions (30). Fifty regions were defined using Ward clustering (R i386 3.1.0, “Rcmdr” package, “FactoMineR” plug-in) based on the mean annual temperature and the annual temperature range (Worldclim, resolution of 1 km, monthly mean from 1950 to 2000). Environmental variables are calculated at the landscape level and at the regional level (ArcGIS 10.1 and FRAGSTATS, version 4). The regional level is only used in the multilevel model. Specific hypotheses are made for the effect of environmental variable at the landscape and regional levels.

**Table 1:** **Variables and their hypothesized effect (X) on the bank voles’ abundance, virus *ex vivo* survival, and human repartition (numbers in brackets indicate references)**.

Variables	Bank voles	Virus	Human	Resolution	Units	Sources
Annual precipitation (8, 23)	X	X		1 km, 1950–2000	mm	Worldclim
Maximum temperature in summer (8)		X		1 km, 1950–2000	°C	Worldclim
Minimum temperature in winter (8, 9, 13, 19)	X	X	X	1 km, 1950–2000	°C	Worldclim
Snow cover (8, 14–18, 22)	X	X		0.05°, 2000–2008	Area percentage	MODIS
Proportion of forest	X			100 m	Area percentage	Corine 2006 (EEA)
Proportion of coniferous forest (25)	X			100 m	Area percentage	Corine 2006 (EEA)
Proportion of broadleaved forest (24)	X			100 m	Area percentage	Corine 2006 (EEA)
Proportion of mixed forest	X			100 m	Area percentage	Corine 2006 (EEA)
Forest contiguity index (6, 7)	X			100 m	None	Corine 2006 (EEA)
Built-up areas in forest ecotones (24, 27)	X		X	100 m	Area percentage	Corine 2006 (EEA)
Enhanced vegetation index (EVI) (28)	X			0.0083°, 2001–2012	None	MODIS
Number of green days (28)	X			0.005°, 2006–2010	Number of days	MODIS
Soil water index (SWI) (8, 23)	X	X		25 km, 2007–2010	None	TU-WIEN
Population proximity index (31)			X	0.0083°, 2005	Number of persons	Environment Research Group Oxford

##### Climatic variables

Climatic variables are assumed to have the same effect at both levels. Higher mean annual precipitations (Worldclim, resolution of 1 km, monthly mean from 1950 to 2000) are assumed to reflect higher air humidity and should increase the probability of presence of NE cases. The mean maximum temperature in the summer (Worldclim, 1 km, monthly mean from 1950 to 2000) is calculated to identify the driest and most unfavorable environments for virus *ex vivo* survival. Summer is defined as the months of June, July, and August. In some areas, this can reflect more human outdoor activities. The mean minimum temperature in the winter (Worldclim, 1 km, monthly mean from 1950 to 2000) is added to our database. Winter is defined as the months of December, January, and February. Lower minimum temperatures in winter are assumed to increase the presence of NE cases (thanks to better virus *ex vivo* survival) as well as the range of minimum temperature suitable for more human outdoor activities. Snow cover is here measured by the annual mean percentage of pixel area covered by snow (MODIS, 0.05°, 2000–2008 monthly mean). Less extensive snow cover (increasing human exposure) and extensive cover (favoring bank voles and virus *ex vivo* survival) should increase the probability of NE cases presence while very extensive cover (reflecting harsh condition) should decrease the probability.

##### Land use variables

We hypothesize that the proportion of forest [Corine 2006 (European Environment Agency, EEA), vector map] indicates the presence of bank voles at the landscape level, and the presence and also the abundance of bank voles at the regional level. The proportions of coniferous, broadleaved, and mixed forest [Corine 2006 (EEA), vector map] are used. It is assumed that broadleaved forest is preferred over coniferous forest but the effect of forest type may differ between the landscape and regional levels. Indeed, if a region is characterized by a large proportion of coniferous forest, the proportion of broadleaved forest could be significant at the landscape level but not at the regional level as, regionally, coniferous forests are used by default by the bank vole. The contiguity index of forest patches [Corine 2006 (EEA), vector map] is used as a proxy to represent contacts between rodents, increasing prevalence in bank voles and so, NE cases presence. At the landscape level, a higher proportion of built-up areas in a buffer of 150 m around forests [Corine 2006 (EEA), vector map] would indicate a higher probability of contact between bank voles and humans. At the regional level, high levels of urbanization may nonetheless limit PUUV transmission.

##### Vegetation indices

The mean enhanced vegetation index (EVI) (MODIS, 0.0083°, 2001–2012 mean) and number of green days per year (MODIS, 0.005°, mean from 2006 to 2010) are included. Green days represent the number of days between the detection of the start of a growth cycle and the end of the cycle, when vegetation greenness decreases. These indices are hypothesized to reflect food availability and the number of litters (more food may reflect smaller territories and more nests) at the landscape level, as well as a longer breeding season at the regional level, and should thus increase the disease presence probability at both levels.

##### Soil variable

Higher soil water index (SWI) (TU-WIEN, 25 km), averaged over 2007–2010, should improve the virus *ex vivo* survival at the landscape level. At the regional level, we assume that a higher soil water index gives information about land use (e.g., presence of marshes and bogs) unfavorable for the bank voles.

##### Human distribution variable

The probability of human NE cases increases with human population density, whether at the landscape or regional level. To reflect the presence of humans, the mean distance weighted population proximity index (Environment Research Group Oxford, 0.0083°, 2005) is considered. The population proximity index represents the population likely to visit a place, taking into account the population in the surrounding environment (31).

### Methods

#### At which levels do the variables operate and how do they influence NE distribution?

The spatial distribution of NE cases in Europe is first modeled using multilevel logistic regression in order to identify significant environmental variables at the landscape and regional level. The intraclass correlation coefficient, a measure of the proportion of the variance found at the regional level (32, 33), is first calculated. If this value is high, a multilevel approach is recommended. A full multilevel model is subsequently fitted (32).

Multilevel regressions allow including intercepts varying between regions (random intercept model) and/or slopes varying between regions (random slopes model). Variables at the landscape and regional level are tested in a model with random intercepts and a model with random intercepts and slopes (“lme4” package in R2.12.0). These models are compared with an empty model and with each other using an ANOVA test based on the Akaike information criterion and the Bayesian information criterion. The final model includes the variables significant in regressions with one explanatory variable and the random intercepts and slopes, which have better Akaike and Bayesian information criterion. To avoid collinearity issues, variables with a variance inflation factor >10 are removed. The area under the curve (AUC) is used to evaluate the predictive power of the model (34). Resulting probabilities of being in presence of a case and false presences and absences are mapped.

#### What are the effects of environmental variables across European ecoregions?

**Figure 2:**
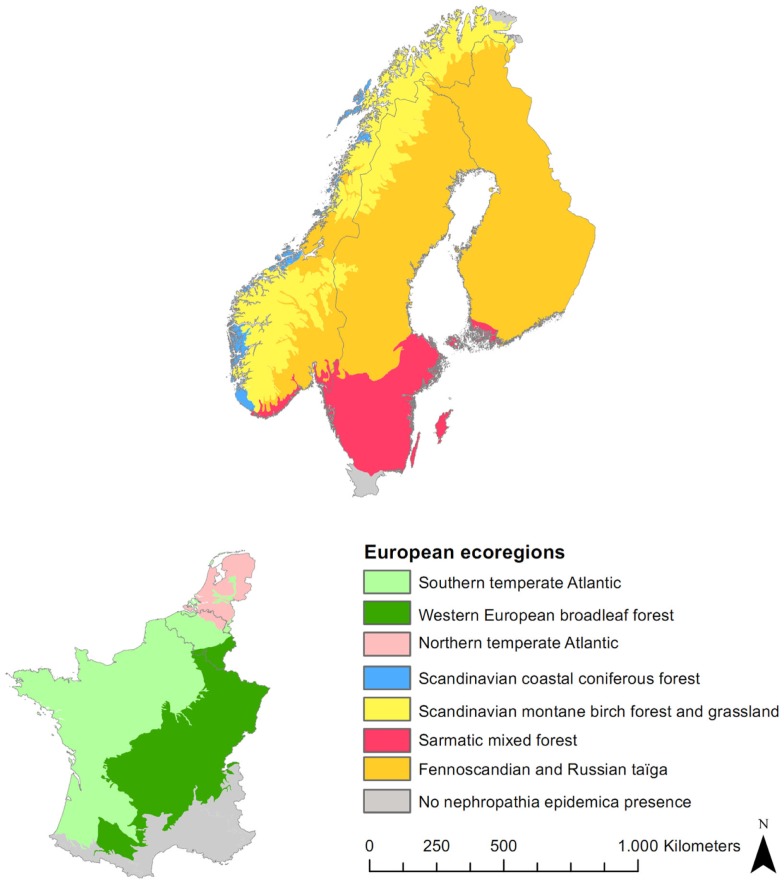
**Digital map of European ecological regions for the study area**.

In order to better distinguish the effects of environmental variables across Europe, logistic regressions are fitted for each ecoregion using landscape level variables. Seven ecological regions encountered in the study area are compared (European Topic Centre on Nature Protection and Biodiversity) (Figure 2).

The effect of environmental factors is not necessarily linear, and may be positive in some regions and negative in some other. Because of the broad range of values covered, this is likely to be encountered in our study area. This type of relationship is poorly addressed by classical regression, and a niche model is thus needed to explore the effect of the landscape level variables across the study area.

**Figure 3:**
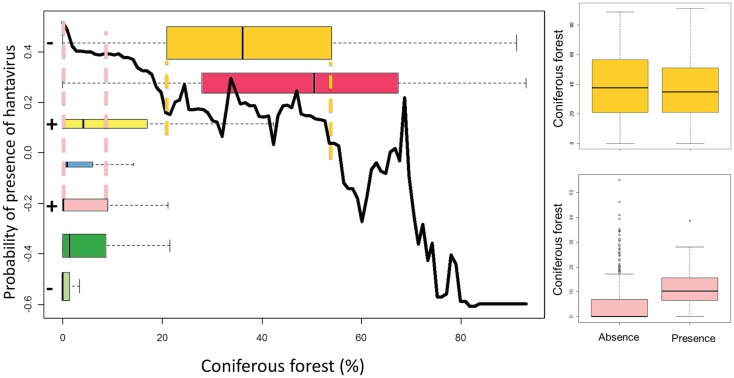
**LEFT – Black curve: relative probability of presence of hantavirus human cases according to the evolution of the variable as modeled by BRT**. Colored boxplots: distribution of the variable by ecoregions (colors refer to Figure 2 and the height of the boxplot is proportional to the number of points by ecoregion). + and – signs: sign of the logistic regression coefficient, if significant, for this variable in this ecoregion. Dashed color lines: boxplot extent (second to third quartile) of an ecoregion on the black curve. RIGHT – Boxplots of the presence and absence distributions of coniferous forest for two ecoregions.

Boosted regression trees (BRT), which allow to model non-linear response, are used to build an European model including all variables (“gbm” package in R) (35). The AUC is here based on cross-validation on 10 subsets, as overfitting is a known issue with BRT. The relative importance of a variable, a weighted measure of the number of times a variable is used to build the consecutive trees, is calculated. Graphs of the probability of presence of the disease as a function of the value of the variable are produced. Only the global trends should be considered, as local peaks may result from interactions with other variables (e.g., strong interactions between the built-up areas in forest ecotones and the maximum temperature in summer are observed). Boxplots representing the distribution of the variable in each ecological region, with colors corresponding to the map in Figure 2, are superimposed to this curve in order to jointly examine the global effect as identified by BRT and its subset in the variable range of each ecoregion. Finally, the signs of the significant coefficients (at the level of 0.05) of bivariate logistic regressions by ecoregion are added in front of each boxplot for comparison with the trend modeled by BRT in this ecoregion. An example graph is presented in Figure 3.

## Results

### At which levels do the variables operate and how do they influence the NE distribution?

The intraclass correlation coefficient shows that 57% of the variance is at the regional level. A multilevel model is therefore advised. Following the variance inflation factor analysis, snow cover, green days, and the proportion of forest are removed from further analyses to avoid collinearity issues.

In bivariate analyses, all variables at the landscape and regional levels are highly significant, except the maximum temperature in summer and the EVI at the landscape level.

The results of the multilevel logistic regression are presented in Table 2. The AUC is 0.895 ± 0.004 and indicates a good predictive power.

**Table 2:** Multilevel logistic regression (significant at the level of *0.05, **0.01, and ***0.001).

	Landscape level			Regional level		
	Estimator	*P*-value	Random slope	Estimator	*P*-value	Random slope
Annual precipitations	0.00	2.23E–01				
Maximum temperature in summer				−0.94**	2.34E–03	
Minimum temperature in winter	−0.15*	3.34E–02		−1.62*	2.69E–02	Yes
Coniferous forest	−0.00	2.74E–01	Yes	−0.10	1.75E–01	
Broadleaved forest	0.02	5.99E–02	Yes			
Mixed forest	−0.01	6.97E–01	Yes	−0.39	9.87E–02	
Contiguity of forest	0.01	9.61E–01	Yes	37.66*	1.26E–02	
Built-up areas in forest ecotones	0.28***	1.41E–15	Yes			
EVI				0.04*	2.46E–02	Yes
SWI	0.00	2.07E–01		−0.14*	2.01E–02	
Population proximity index	0.01	1.42E–01	Yes	0.11*	4.44E–02	

Random slopes are included for the landscape level variables coniferous forest, broadleaved forest, mixed forest, contiguity of forest, built-up areas in forest ecotones, and population proximity index and, at the regional level, minimum temperature in winter and EVI.

At the landscape level, the coefficient of the variable built-up areas in forest ecotones is significantly positive at the level of 0.001. The coefficient of the minimum temperature in winter is significantly negative at the level of 0.05. At the regional level, the coefficients of forest contiguity, EVI, and population proximity index are significantly positive at the level of 0.05. Also, the maximum temperature in summer is negatively significant at the level of 0.01, and the minimum temperature in winter and SWI at the level of 0.05.

**Figure 4:**
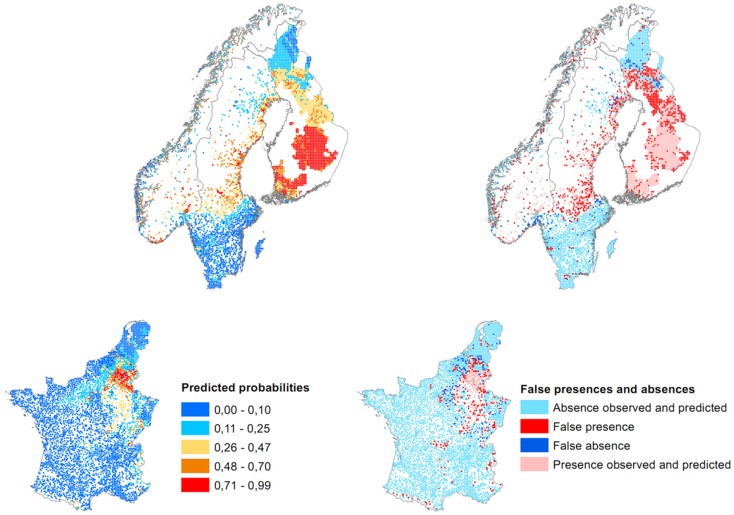
**Map of predicted probabilities and false presences/absences of the European multilevel model (threshold for presence = 0.25, when the sensitivity equals the specificity at 84%)**.

The predicted probabilities and the false presences/absences of the model are mapped in Figure 4. The model predicts the highest concentrations of probabilities in northeastern France, southern Belgium, inland Norway, central Sweden, and southern and middle Finland. False presences are mainly located in areas where the diseases is already established and along the Norwegian coast. A few false presences are also predicted in less expected areas: southern and central France and southern Sweden. False absences are more present at the western and southern border of the French disease distribution, in northern Belgium, in the southern Netherlands, in southern and northern Sweden, and in northern Finland.

### What are the effects of environmental variables across European ecoregions?

The multilevel regression indicates that the effect of environmental factors varies across regions and random slopes were included for several variables. Logistic regressions are therefore elaborated for each ecoregion (Figure 2). When significant, forest, mixed forest, forest contiguity, and built-up areas in forest ecotones are positive; EVI, green days, and population proximity index are positive or negative; annual precipitation is positive except for the Scandinavian montane birch forest and grassland; maximum temperature in summer is negative except for western European broadleaf and sarmatic mixed forest; minimum temperature in winter is negative except for Fennoscandian and Russian taiga; snow cover and the SWI are positive except for Fennoscandian and Russian taiga; coniferous forest is negative except for the northern temperate Atlantic and the Scandinavian montane birch; and broadleaved forest is positive except for northern temperate Atlantic and Fennoscandian and Russian taiga.

The AUC of the boosted regression trees model is 0.921 ± 0.003. The AUC on cross-validation is 0.893 ± 0.003. These indicate a very good predictive power of the model. The variables with a relative importance over 10 are the minimum temperature in winter, the snow cover, and the maximum temperature in summer.

**Figure 5:**
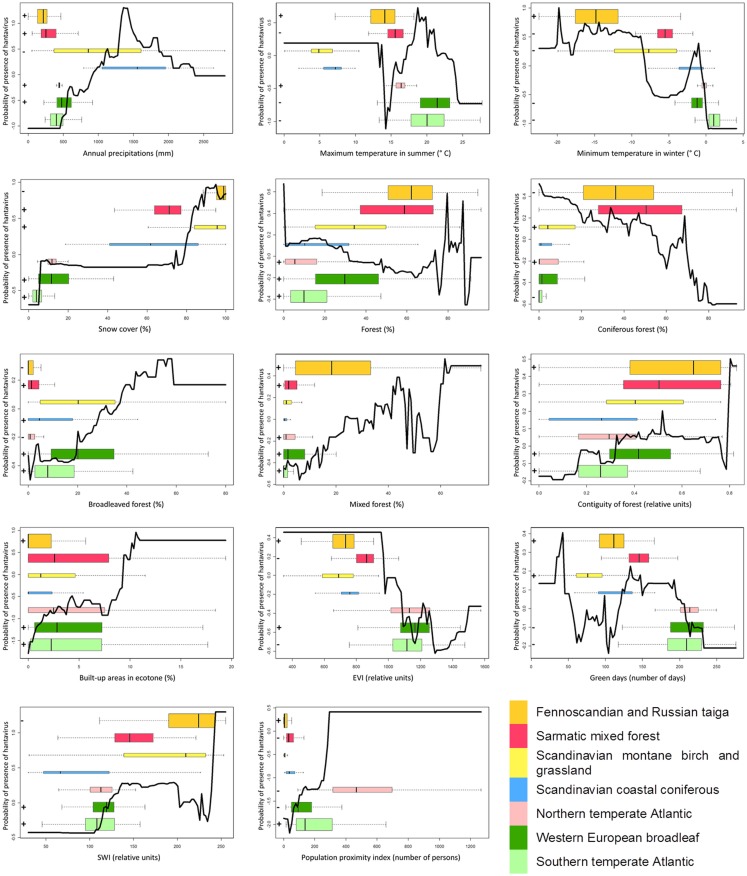
**Response curves of the variable according to the predicted probabilities of the boosted regression trees, boxplots of the variable per ecoregion, and significant signs of the coefficient of bivariate logistic regressions per ecoregion**.

The response curves (Figure 5) of snow cover, broadleaved forest, mixed forest, contiguity of forest, built-up areas in forest ecotones, SWI, and population proximity index show an overall positive trend. The curves of the minimum temperature in winter, forest, coniferous forest, and EVI present an overall negative trend. Green days show a mixed curve. Annual precipitation and maximum temperature in the summer display an increasing then decreasing trend in probabilities.

The boxplots in Figure 5 indicate that variables with a similar width of boxes between regions are the proportion of mixed forest, the built-up areas in forest ecotones, and the population proximity index. Variables with boxplot extents following a gradient from South to North are minimum temperature in winter, snow cover, contiguity of forest, and the SWI. The ecoregional boxplot of EVI and green days shows a strong difference between western European and Fennoscandian ecoregions. Finally, the boxplot extent seems mixed between regions for the annual precipitations, the maximum temperature in summer, forest, coniferous forest, and broadleaved forest.

The general trend by ecoregions can be compared to the sign of the coefficient from the bivariate logistic regressions (Figure 5). The signs of the regression coefficient and the BRT trends are consistent for minimum temperature in winter, snow cover, mixed forest, contiguity of the forest, built-up areas in forest ecotones, green days, and SWI.

## Discussion

### Large-scale modeling

**Figure 6:**
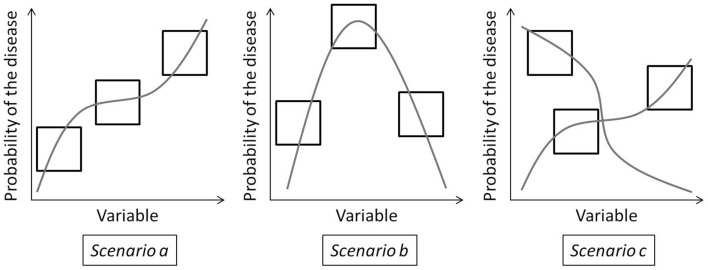
**Three scenarios that can happen when modeling variables over a large study area, squared frames represent different subregions of a large study area**.

Both the multilevel and the BRT models have very good predictive powers. The distribution of zoonoses at a large-scale using a single database built from national databases is successfully modeled. However, large-scale models can obscure the local effect of environmental factors or how they may differ from place to place. With these models, the interpretation of the variables may generate misleading conclusion as wide range of conditions cannot be summarized into one explanation. We identify three scenarios of response to a variable that can be encountered in modeling studies at a large-scale (Figure 6). These scenarios are illustrated by variables from this study, but are relevant to other disease modeling studies.

#### Scenario A

The first easiest scenario implies that a variable has the same effect over the complete study area. These variables can be easily included in a large-scale model. In our study, the built-up areas in forest ecotones are assumed to increase the human exposure. This is reflected both by the ecoregional regressions and the global BRT model.

#### Scenario B

The second scenario, the most common, is when the variable has a non-linear effect on the probability of the disease. Because of this, the effect of the variable varies from region to region, depending on the value of the variable encountered. For these variables, results and hypotheses from local studies can be used for places with the same variable extent. They can be used in non-linear large-scale models. For example, looking at the snow cover BRT trend, less extensive snow cover (such as in southern Europe) increases the probability as it relates to more contact between the virus/bank voles and humans; a more extensive snow cover increases the probability even more as that implies a larger bank voles population and a better *ex vivo* virus survival; very extensive snow cover (such as in the extreme north of Europe, in Fennoscandian, and Russian taiga) has a decreasing probability because of the harsh conditions for the bank voles and the increasing pressure due to lower prey diversity. The sign of the ecoregional regression matches these hypotheses as well as the BRT response trend.

#### Scenario C

The third scenario, the most difficult to identify, is when the variable does not seem to reply to one hypothesis, whatever the extent of its distributions. Different hypotheses are needed for each region. For these variables, results from local studies cannot be applied and interpolated to other places and their interpretation in large-scale study may lead to misleading conclusion. Taking the coniferous forest as an example, the global BRT trend is negative as it is generally expected, but the sign of the regression coefficient appears significantly positive for the northern temperate Atlantic and the Scandinavian montane birch ecoregions. In the northern temperate Atlantic ecoregion, there are very few forests and it is possible that bank voles are found in coniferous forests, lacking a better habitat. For the Scandinavian montane birch ecoregions, coniferous forests are in valleys while mountains are colonized by birches (36). Soils on the slopes are drier and know important runoff. In those areas, the conditions in broadleaved forest are less favorable for the virus and rodents than in the coniferous forest. This is probably why coniferous forests appear to be positively related to NE presence in these regions. The effect of coniferous is thus not the result of one non-linear process but can be explained by the local particularities of ecoregions. These particularities may result from the level of the other variables or from historical and cultural factors.

### Model variables

So far, the distribution of NE cases has not been studied at the European level, and this study helps to answer whether factors impact the distribution in one region or at a multi-country level [see question raised in Ref. (11)]. Our model highlights the new finding that climatic variables as well as the connectivity of forests act more at the regional level than at the landscape level. The multilevel logistic model shows that NE cases are mostly found in populated regions with connected forests, high EVI, and low SWI. These regions have a low maximum temperature in summer and a low minimum temperature in winter (summers are mild and winters are cold). In these regions, landscapes with a higher proportion of built-up areas in forest ecotones and a lower minimum temperature in winter are expected to be more at risk. Models results are consistent with what has been already hypothesized on NE in Europe (2, 11). Unexpected curves are nonetheless observed for EVI and green days but their relative importance is low, and their effect complex to interpret (37).

By combining ecoregional regressions and a global BRT model, we identified variables for which it is advised to pay attention when hypothesizing effects (Scenario C): precipitations, coniferous forest, broadleaved forests, and population. For the minimum temperature in winter, the snow cover, green days, the SWI, the proportion of mixed forest, the built-up areas in forest ecotones, and the contiguity of forests, our results suggest that they follow one process that can be non-linear.

### Model predictions

The predicted probabilities map shows that, according to environmental data, fewer cases should be expected in southern, western, central, and the extreme east of France, northern Belgium, the Netherlands, southern and northern Sweden, and northern Finland. The BRT model yields similar predictions. Also, a lot of false presences are found in areas where the disease is already recorded and false absences are located at the edge of these areas. If variables stay at their current values (this model predicts the current situation and not the future), false presences can be interpreted as suitable places to find the disease and where the disease may appear in the future, while false absences can represent unsuitable environments. It seems that the recorded data already covered the potential distribution of NE cases. A spread of the disease should not occur but intensification is possible.

### Limitation of the models

As we learned here, effects can vary between scales and, even if the landscape scale is interesting, a finer scale may bring further details and reflect more particularities. Exact geographical coordinates are rarely known or disclosed, due to privacy rules but also due to the nature of the hantavirus pathogenesis, and the size of the administrative units may be too big to make proper models. NE cases are not reported in the same way everywhere (between and within countries). Cases can, for example, be identified from antibodies from serological surveys or by the identification of clinical symptoms. A bias may probably result from the difference of these reports. Inclusion of a variable accounting for these differences could solve this issue but is particularly challenging as distinction of the way of records is not always known. Cases are most often reported by residence or where the disease is identified, rather than the true infection location. Therefore, some presences are reported in unexpected places, like in southern Sweden where PUUV is reportedly absent. These presences were nevertheless included in the database and were all predicted as absences. However, in this area, some false presences are predicted near these false absences. It could indicate that the recorded presences do indicate local transmission, unless these were indeed false predictions. The models presented here are not designed to predict the future distribution of PUUV. False presences indicate places where the confirmation of the absence or presence of cases must be assessed (e.g., through serosurvey among exposed humans). In southern and central France, absence of confirmed case will probably result from the fact that places are too far and isolated from the current distribution to imagine that the disease will reach these places. The other false absences places like southern Sweden, the Netherlands, and the Norwegian coast, even if located in lower probability areas, should be carefully considered in the future for monitoring and surveillance.

## Conclusion

This study successfully models the pan-European NE distribution using a compilation of national disease data source and European level environmental databases. Europe covers diverse landscapes and ecoregions and, even if some variables are recurring in the literature for various areas, the underlying hypotheses can be different. This study highlights that these differences can be the result of local specificities or of the non-linearity of the processes. When the effect of a factor varies locally, its inclusion in a large-scale model is compromised.

According to both multilevel logistic and non-linear model, the distribution of PUUV seems limited in space by environmental variables that are identified here. Our model does not predict an important spread of the disease, assuming current conditions, but possibly intensification in places where the disease is already there. Also, some lower probabilities areas such as the South of Sweden, the Netherlands, and the Norwegian inner coast should be further studied.

## Author Contributions

CZ and SV contribute to the conception and design of the work, the analysis and interpretation of data, and the drafting of the work. SQ, HH, OL, OV, J-MR, CR, AS, KV, and MH contribute to the design of the work, the acquisition the data, the critical revision for important intellectual content. All the authors approve the version to be published and agree to be accountable for all aspects of the work in ensuring that questions related to the accuracy or integrity of any part of the work are appropriately investigated and resolved.

## Conflict of Interest Statement

The research was conducted in the absence of any commercial or financial relationships that could be construed as a potential conflict of interest.

## Supplemental Materials

The Supplementary Material for this article can be found online at http://journal.frontiersin.org/article/10.3389/fpubh.2015.00054/abstract

One Excel file with three sheets:

1. Table with results for each ecoregion of logistic regression in bivariate, complete, and step models.
2. Table with results for each country of logistic regression in bivariate, complete, and step models.
3. Table of random intercepts and slopes added in the multilevel model.
